# The technology of tongue and hard palate contact detection: a review

**DOI:** 10.1186/s12938-021-00854-y

**Published:** 2021-02-06

**Authors:** Syatirah Mat Zin, S. Z. Md Rasib, Fatanah M. Suhaimi, M. Mariatti

**Affiliations:** 1 Craniofacial and Biomaterial Sciences Cluster, Advanced Medical and Dental Institute, Universiti Sains Malaysia, 13200 Kepala Batas, Penang Malaysia; 2 School of Materials and Mineral Resources Engineering, Universiti Sains Malaysia, Engineering Campus, 14300 Nibong Tebal, Penang Malaysia

**Keywords:** Electropalatography, Speech pathology, Medical technology, Speech rehabilitation

## Abstract

The tongue and hard palate play an essential role in the production of sound during continuous speech. Appropriate tongue and hard palate contacts will ensure proper sound production. Electropalatography, also known as EPG, is a device that can be used to identify the location of the tongue and hard palate contact. It can also be used by a speech therapist to help patients who have a speech disorder. Among the group with the disease are cleft palate, Down syndrome, glossectomy, and autism patients. Besides identifying the contact location, EPG is a useful medical device that has been continuously developed based on the patient’s needs and treatment advancement. This article reviews the technology of electropalatography since the early introduction of the device. It also discusses the development process and the drawbacks of the previous EPG systems, resulting in the EPG’s upgraded system and technology. This review suggests additional features that can be useful for the future development of the EPG. The latest technology can be incorporated into the EPG system to provide a more convenient method. There are some elements to be considered in the development of EPG’s new technology that were discussed in this study. The elements are essential to provide more convenience for the patient during speech therapy. New technology can accelerate the growth of medical devices, particularly on the development of speech therapy equipment that should be based on the latest technological advancements available. Thus, the advanced EPG system suggested in this article may expand the usage of the EPG and serve as a tool to provide speech therapy treatment services and not limited to monitoring only.

## Background

EPG is a computerized system that can record tongue and palate activity in the production of speech during real-time. EPG has gained wide acceptance in speech pathology and linguistic research. This paper reviews the EPG system and the use of EPG from the year 1976 to 2020. In particular, this study aimed to examine the design and development process of commercially available EPG systems since its early introduction. This article highlights the history of EPG and the importance of EPG in speech therapy. It can be summarized that EPG's future technology should consider improving the drawback of the current EPG. Additionally, the essential elements of future EPG are also suggested in this article.

## Overview of EPG

Speech disorder assessment is commonly conducted using an auditory perception, which is based on the anatomical and physiological knowledge of the tongue movement of speech–language therapists (SLTs). During a treatment, SLTs have to imagine the tongue movement from one position to another during continuous speech production. An experienced SLT is required to provide speech analysis using this technique [1]. Due to the nature of the auditory perception technique, the speech therapy process, particularly in some countries, is not thoroughly comprehensive because of the shortage of experienced SLTs [2]. However, a technique was introduced in the year 1970 to identify tongue and hard palate location known as electropalatography (EPG).

EPG provides the recording of dynamic speech features, and thus enables the detection of sound production. At the same time, the movement of the tongue and hard palate contact can be identified from the EPG patterns [3]. EPG is used to diagnose and analyse the tongue and hard palate contacts pattern during continuous speech production in real-time [4]. Each consonant produces during a continuous speech has its unique characteristic of contact pattern. The essence of the contact pattern depends on the location of the tongue and hard palate contact [5]. The location of the tongue and hard palate contact is detected using electrode sensors that are embedded on an artificial palate. These electrode sensors detect the contact and send the signal to a computer.

In addition, EPG is an established instrument used in phonetic and clinical research [6]. Several studies proved that EPG is a highly useful tool for speech research, diagnosis, and treatment of a range of speech disorders [7]. From 1970, there were three commercial companies involved in manufacturing the artificial palate, which was Rion Co Ltd, Japan (Rion system), Kay Elemetrics, USA (Kay Palatometer), and Reading University (Reading system) [8]. Complex and high production cost of the palate caused a number of palate modifications to be discontinued, such as the Rion electrograph (DP-01) in 1990 [9], and the Palatometer produced by Fletcher et al. in 1988 [10]. Currently, there are three commercially available companies, which are:

1. SmartPalate system (CompleteSpeech), formerly known as Logometrix.
2. LinguaGraph (Rose Medical Solution) and
3. WinEPG (Articulate Instruments Ltd).

SmartPalate system developed from the Kay Palatometer initially sold by Kay Elemetric was discontinued after Professor Fletcher decided to build his own company. Under LogoMetrix Corporation, the research and development were continued, and the company changed to CompleteSpeech, and SmartPalate became one of the products [11]. In the LinguaGraph system, the Reading palate is connected to the LinguaGraph unit, worn around the neck [8]; whereas, in the WinEPG system, the Reading palate and Articulate palate were connected with the WinEPG measurement hardware manufactured by the Articulate Instruments Ltd [4].

**Fig. 1:**
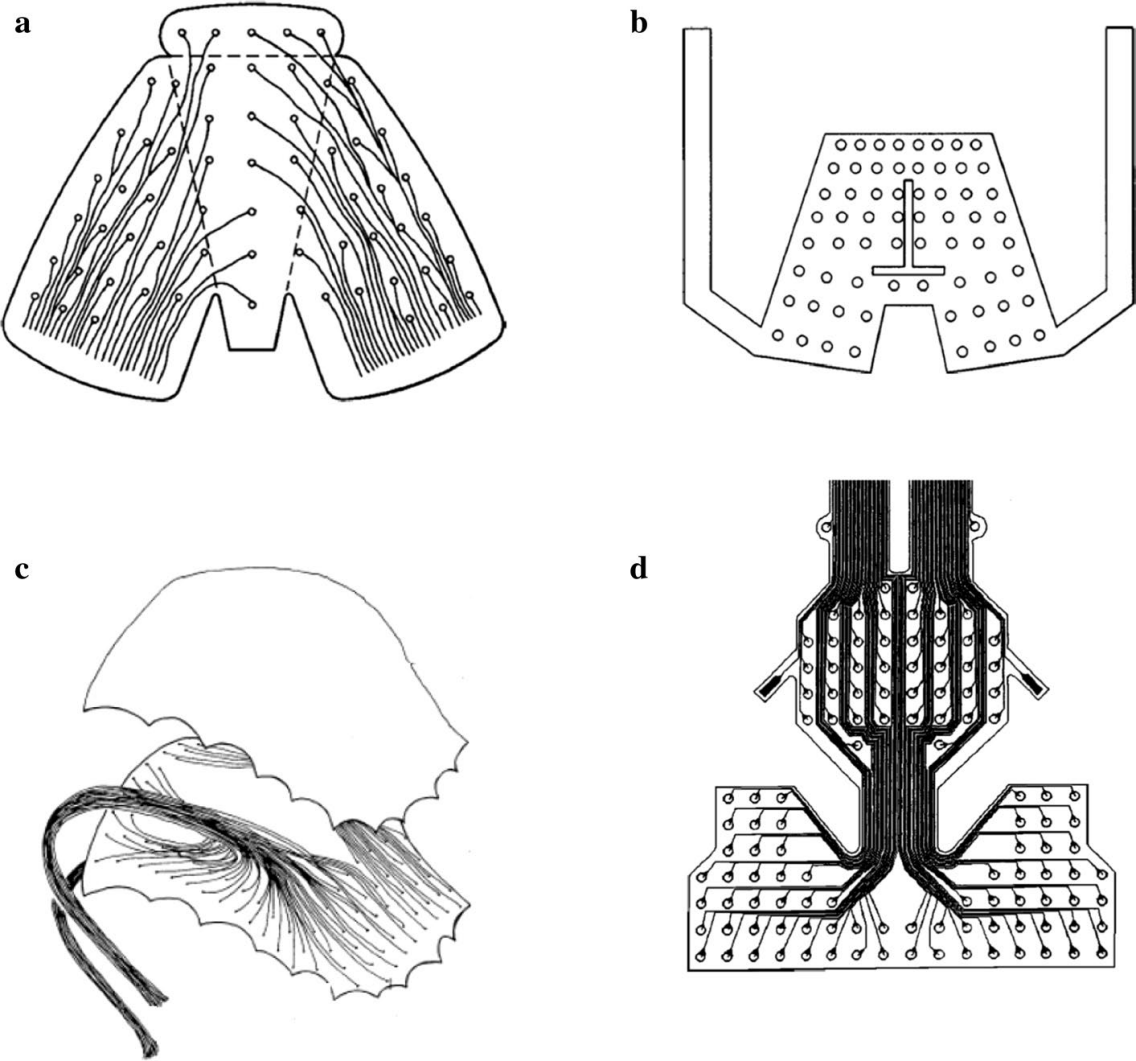
Modification and development of **a** flexible circuit of frame body artificial palate by Rion Co Ltd, **b** flexible circuit of T bar cut-out introduced by Hardcastle et al., **c** development of palatometer by Fletcher et al. in 1978, and **d** flexible circuit three-lobed structure by Fletcher et al. 2005 (**a**, **b**, and **d** reproduced with permission by Wrench et al. [3] **c** reproduced with permission by Fletcher et al. [17])

Clinical and ongoing studies on patients have improved EPG to make it more convenient, cheaper, and more accurate. Starting from the production of flexible circuits in the 1970s, which is patented by the Rion Co Ltd, the flexible circuit productively reduced the manufacturing time and cost. This flexible circuit is much more effective than the traditional Reading system and Palatometer that are made by rigid acrylic and acrylic vacuum as a thin sheet material shape where the electrode contact is embedded in the acrylic resin [4]. Table 1 shows the general timeline of EPG until the latest modification. After the Rion Co Ltd patented the flexible circuit board (Fig. 1a), Rion Co Ltd more focussed on the improvement of display device by introduced a signal-receiving electrode from the linguapalatal contact pattern with a preliminarily set standard linguapalatal contact pattern for specific phonation [12]. Rion Co Ltd then extended the EPG to transmitted linguapalatal contact pattern as electromagnetic waves and displayed as dynamic patterns [13].

**Table 1:** Timeline of modification and development of EPG

Year	Modification and development of EPG	Refs
1974–1978	Europe, EPG developed at Reading University	[14, 15]
1979	Japan, the Rion Co Ltd patented a flexible insulative circuit board and improvement in the fabrication method	[16]
1978	US, Development of Palatometer by Fletcher	[17]
1981	Japan, the Rion Co Ltd patented EPG by introducing a single signal-receiving electrode by a single detector	[12]
1982	The Rion Co Ltd again patented their EPG by improving transmission and receiver from the EPG to the display device	[13]
1989	T-shape cut-out by Hardcastle et al. with flexible circuit design	[4]
1991	The Reading palate improvement in hardware and software design	[18]
2005	Fletcher produced patented three-lobed structure	[19]
2007	Wrench developed and patented articulate-style palate	[3, 20]
2014	Ghovanloo et al. patented wireless Real-time Tongue Tracking	[21]
2018	Development on SmartPalate	[11]

The use of flexible devices is continued by Hardcastle et al. in 1989 [4], where the ‘T' bar cut-out in the centre has been introduced, as shown in Fig. 1b. 31 tracks from each electrode (31 tracks at left and 31 tracks at right) connected separately and exit at the corner of the mouth. A double-sided copper plate was used to avoid short circuits between the tracks. An insulating layer is placed in between two copper surfaces; hence some tracks can be imprinted on both surfaces. However, Hardcastle et al. [18] then declared that there are disadvantages in this flexible EPG pattern. Some of the disadvantages are that the sharp pattern and design are difficult to fit in the user's mouth, and the flexible palate material is still thick and uncomfortable. Hardcastle et al. then only made some improvements in software and hardware with the recent version of the Reading system (EPG3).

The conventional palatometer (Fig. 1c) patented by Fletcher et al. in 1978 [19] that uses thin sheet material with the electrode located by melted of copper into punched holes was improved by using a flexible circuit with electrode combining with nasometer apparatus patented in 2005. The flexible circuit contains three intercoupled lobes, such as a butterfly shape for electrodes replacement and formed in a concave configuration, as shown in Fig. 1d. These unique lobes make it suitable to all size and shape either smaller or bigger palate. These three-lobe structured palates manufactured starting by taking an impression of the user, followed by constructing a stone model based on the impression. A base plate made by soft plastic material is then prepared by the stone model’s thermoforming process to form a palatal body. After several cutting processes and conforming the shape to the patient, a flexible printed circuit containing at least 110 sensors is attached using adhesive such as cyanoacrylate adhesive to the base plate. Some adjustments are made to conform to the user’s shape and to ensure the palate fits the user. The flexible circuit is connected to a processing and display equipment by leads containing electrical connectors.

Unlike the other palate design, this three-lobe structure palate did not wrap the leads from the back, but directly out the front of the flexible printed circuit. Two embodiments are prepared with a similar manufacturing process, as mentioned earlier, and the configuration is displayed in split-screen [19]. One of the screens represents contact from the electrode sensor by the user, while another screen is generated from computer software or another user [22]. One disadvantage of this design is that the ready-to-use flexible circuit with the permanently sited on the strip does not precisely follow the individual user’s palatal. Although the electrode strip can be adjusted, the flat strip is fixed. Based on Schmidt et al.’s study with varying speech sound disorders, all the users successfully sit for the test. However, the same problem due to the fixed electrode strip makes it unreliable to develop an individual articulatory pattern that suits the individual condition [23]. Research and development of the palate were then continued by CompleteSpeech company under the SmartPalate product. The recent related patent (US 2009/0138270 A1) presented that Fletcher et al. keep using the same design by providing guidelines for quantitative accuracy of the speech pronunciation [24].

In 2007, Wrench et al. [3] introduced a new palate design called Articulate palate. The Articulate palate is made after considering many factors concerning the issues from the previous artificial palate (Kay Palatometer and Reading palate) such as contact layout, contact size, material, safety, fit adjustment, lead length, and exit point. The Articulate has attracted interest by improving the quality of speech through the thin palate, and placing contacts at the velar region makes it more reliable. However, some design factors required improvement. They found out that removing the Articulate palate is challenging since the design made is focused on fitting the palate to its place and avoid any movement during pronunciation. The design also should be investigated further as the saliva pooling problem still occurs. Birkholz et al. [25] designed the artificial palate by combining and modifying both Reading and Kay Palatometer palate design using Adam clasps and a cover for the teeth. The acrylic resin that covers molar and premolars was removed and the posterior part is retained using Adam clasps, while the acrylic resin is thermoformed and covered around the incisors and canines to fix the anterior part. Therefore, the idea of this design could be further investigated for the improvement of the Articulate palate.

## Importance of EPG in speech therapy

Speech–language and communication difficulties are common in subjects suffering from speech sound disorder (SSD), auditory processing disorder, Down syndrome, cleft palate, and glossectomy. Some of the cases are due to the anatomy of the tongue and the hard palate of an individual. This is likely to impact daily communication and adversely affect the quality of life [26].

SLTs traditionally used a conventional method, which is auditory-based transcription. During the treatment session, SLTs will ask the subject to produce the sound, which was then recorded. SLTs will replay the sound and teach the subject to produce the correct sound based on the place of articulation of the sound. SLTs must have the knowledge and expertise to determine the articulation place during the production of the sound [1].

EPG has been used for more than 50 years to monitor and improve the articulation patterns of SSD speakers. The effectiveness of EPG in treating an SSD speaker is proven by a study conducted by Carter and Edwards (2004) [27]. There were ten speakers from various backgrounds and ages selected for this study. The finding indicated that the treatment performed for 10 SSD speakers using EPG showed improved sound production [28].

Besides, EPG also helps to improve the production of sound among hearing disorder speakers. A study was conducted to investigate the use of EPG as a therapy tool in enhancing the production of speech for a patient who has a cochlear implant [29, 30]. The finding shows a positive result where there was an improvement in producing velar plosive consonants after 5 weeks of treatment. In placing more emphasis, Bacsfalvi et al. [31] conducted a study to examine the use of EPG for adolescents with hearing impairment. Three subjects diagnosed with severe hearing impairment were chosen to enrol in this program. The remediation treatment has been set for 6 weeks. Their results showed there was an improvement in the articulation influenced by the treatment methodology.

Another type of clinical condition associated with a speech disorder is Down syndrome. Down syndrome is a disorder involving genetic malformation. People with Down syndrome have a problem with physical growth, and speech disorders mainly contributed to the large tongue size [32]. In 1993, a study was conducted to investigate the differences in contact patterns between the tongue and hard palate of a young adult’s speech pattern with Down syndrome [33]. The finding states that people with Down syndrome have unclear articulation during continuous speech. They also have difficulties with phonological delay.

In a similar study, EPG is used as a therapy to reduce speech problems for Down syndrome patients. Besides, EPG is also used to examine the pattern of consonants [ʃ] and [t] for Down syndrome patients [34, 35]. The outcome indicates that many errors that occur during the production of these consonants. Thus, treatment was planned for the patients involving several sessions to obtain a regular pattern for the production of consonants /ʃ/ and /*t*/.

On the other hand, cleft palate patients also used EPG as a treatment for articulation. Cleft palate constitutes a significant health problem. According to Hart et al. [36], between 100 and 500 children born each day all over the world are diagnosed with cleft palate. Cleft palate affects the production of speech, and it will become a significant problem during communication. EPG is also used as a therapeutic instrument in cleft palate patients in several languages, including Japanese [37–39], Cantonese [40], and Swedish [41]. Meanwhile, speech therapy for the cleft patient not only focuses on children, but also on adults. In his study, Fletcher [42] proved that there are differences in speech production and oral motor skill in an adult without palatal corrective surgery.

## Recent development on electropalatograph

### SmartPalate system (CompleteSpeech)

**Fig. 2:**
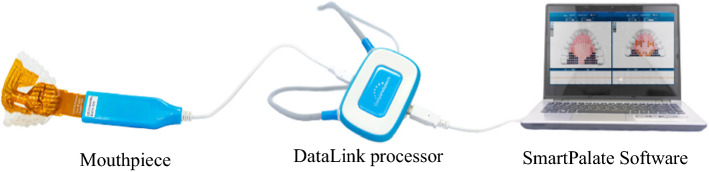
Three components of SmartPalate under CompleteSpeech company (reproduced with permission by CompleteSpeech [11])

The founder of CompleteSpeech, Prof. Samual G. Fletcher, commercialized the SmartPalate as a tool to display tongue to palate contact in real-time as an objective to practise target sound. This artificial palate consists of three main components: a mouthpiece, a DataLink processor, and a SmartPalate Software (Fig. 2). A customized mouthpiece contains 124 gold plate electrodes that captured the tongue placement and sends the tongue to palate contact information to the DataLink processer. SmartPalate software converted the information processed by the DataLink and display the result as a visual representation on the computer screen. This SmartPalate is suitable to be used from children over 8 years to adults to overcome speech barriers such as the pronunciation of ***r***,* l*, ***ch***, ***j***, ***t***, ***d***, ***s***, ***z***, ***k***, ***g***, and, ***sh*** sounds.

### LinguaGraph (Rose Medical Solution)

**Fig. 3:**
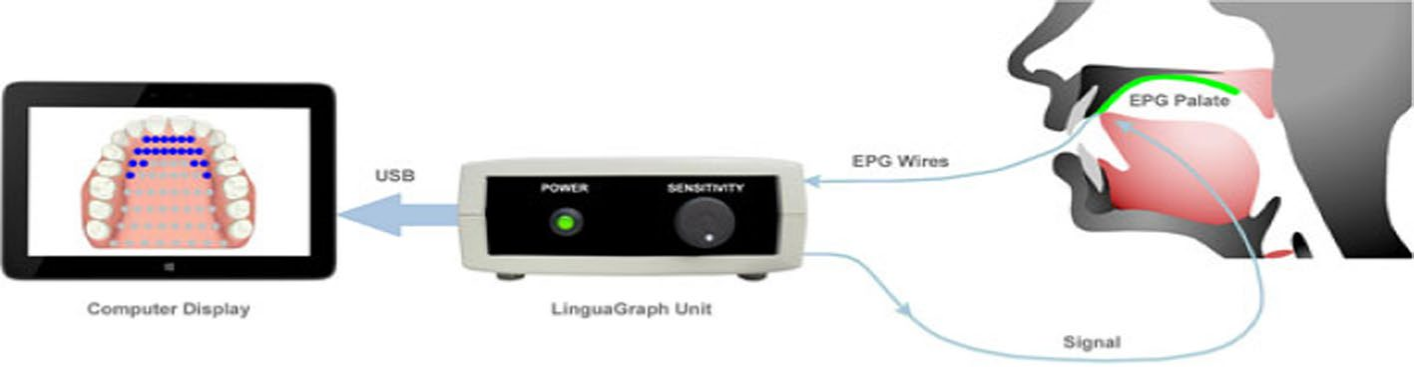
Three component of LinguaGraph under Medical Rose Solution company (reproduced with permission by Medical Rose Solution [43])

LinguaGraph is a commercial EPG system that is user-friendly and suitable for clinical and home-based therapy. Almost similar to the SmartPalate, LinguaGraph also has three components, which are EPG palate, Linguagraph unit, and computer (Fig. 3). The Reading palate is prepared by taking the upper teeth impression of a speaker. A qualified dentist typically takes the upper teeth impression. The impression is then sent either to Rose Medical Solution to prepare the EPG palate or to orthodontic technologists using Electropalatography (EPG) palate kit, which is sold separately by the Rose Medical Solution complete with an instruction manual. The EPG unit is then connected to the LinguaGraph unit to monitor and improve articulation patterns by the adjustable controller to vary the sensitivity of the devices before displaying the pattern on a screen.

### WinEPG (Articulate Instruments Ltd)

**Fig. 4:**
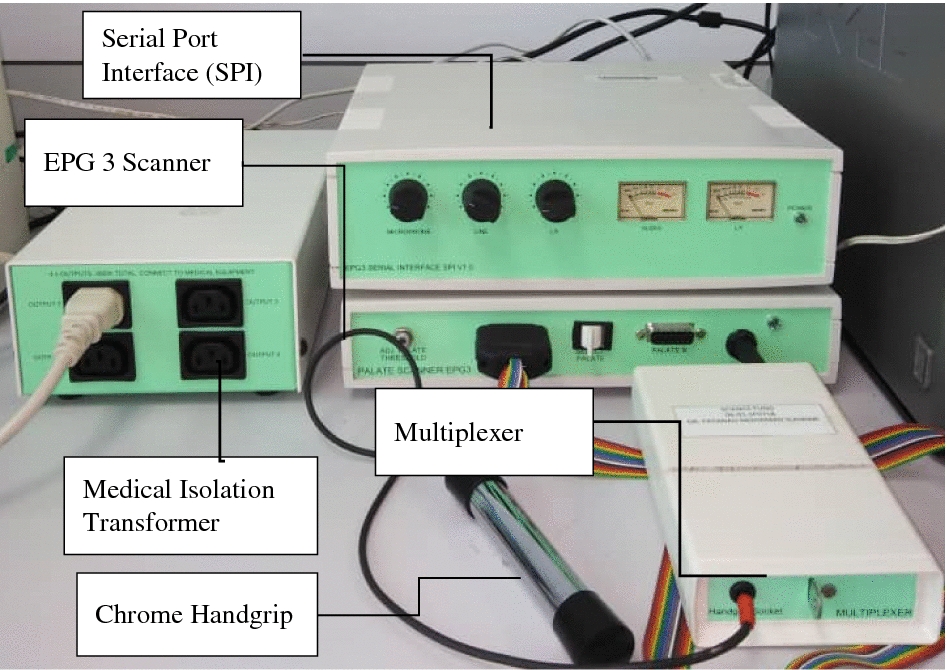
WinEPG hardware setup

Another commercially manufactured EPG system is called WinEPG. WinEPG is a Microsoft window version of an EPG system. The components of WinEPG are more complicated and consist of more than three elements, as shown in Fig. 4. It consists of EPG palate, multiplexer, chrome handgrip, serial port interface (SPI), EPG 3 scanner, and a medical isolation transformer. Reading palate or Articulate palate is used with the WinEPG and connected to the connector slot on the multiplexer, hanging around the user’s neck. Users must hold the handgrip to complete the contact circuit. Once the tongue touched the electrode, a sinusoidal signal from the handgrip is generated from body through the touch tongue and to the electrode. The signal electrode was scanned by the multiplexer and send amplified signals to the main EPG unit. Peaks are detected, and the pre-set reference level is compared to identify the contact made. The signal from the EPG unit was transferred to the computer and display using Articulate Assistant 1.18™. Articulate Assistant 1.18™ makes it possible to analyse the data in real-time.

### Tongue position tracking device (TPTD)

**Fig. 5:**
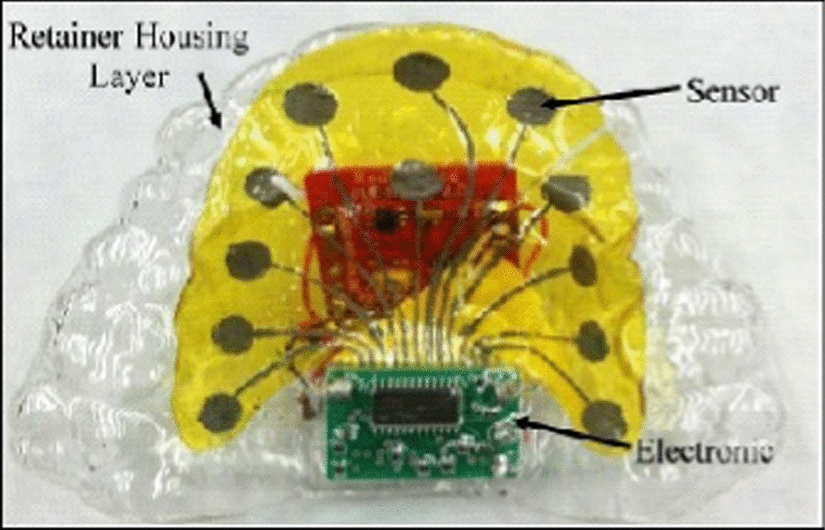
Tongue Position Tracking Device (TPTD) (reproduced with permission by Pastore [44])

TPTD is the recent research development of EPG as an objective to overcome the problem of the previous artificial palate, especially on the wire connection from electrode exit through the mouth to the display instrument shown in Fig. 5. Several attempts have been made in previous design to ensure users feel comfortable and able to pronounce naturally during treatment. One of the efforts to solve the problems is by wrapping the wire directly out to the front instead of wrapping out from the back of the mouth and hanging the wire to avoid pressing tension on the lead. Moreover, the diameter or thickness of tube wire has also been improved using a flexible circuit imprinted with the electrode circuit. Yet there are still barriers from the wire such as the wires should be long enough, and difficult for the user to move freely throughout the treatment process. Thus, it would be best to design an artificial palate in transmitting information wirelessly.

A combination of EPG and glossometry enables TPTD to determine contact patterns and measuring the distance between the tongue and hard palate. This is another advantage of EPG as a method to track any tongue motion that not involve in any contact to the palate when pronouncing phonemes. The TPTD consists of three main components which are retainer housing layer, sensors, and electronic. The retainer is made using specific impression material (Sildent TM Putty) to produce the user’s impression. Unlike the previous palate model, the TPTD palate model uses resin (urethane resin) instead of stone and only took about 1 h to cure. The 0.8 mm of splint material (acrylic thermoplastic) is thermoformed to follow the model shape. Electronic module and electrode are positioned, and again another splint material thermoformed and sealed all the electronic and sensor inside the retainer.

### Pressure mapping with textile sensors

Baldoli et al. [45] conducted an innovation of EPG palate. The latest innovation incorporated textile-based sensing technologies. The prototype comprises 62 piezo-resistive textiles as sensors to detect the tongue and hard palate contact. Besides, the palate was fixed using a glue which is commonly employed as a denture adhesive. This system used Articulate Assistant™ 1.18 as the software to analyse the contact pattern. The reading data was measured by comparing the pressure sensitivity of the sensor. The finding shows that soft sensing effectively measures the tongue and hard palate contact during a continuous speech.

Articles selected to compose this review were gathered from Scopus, PubMed, IEEE *Xplore*, and Science Direct databases. A total of 48 articles and three websites published between 1976 and 2020 were selected in this study. The articles mainly discussed the EPG's design and development and focused on the EPG applications for speech therapy treatment. The use of EPG for speech production in the language was excluded from this study. After reviewing the articles, the future technology of EPG was proposed in this study.

## Future technology of EPG

The design of future EPG is suggested to be flexible, portable, and user-friendly. The new proposed EPG technology is expected to improve the current EPG drawback, especially for a patient who had limited physical movement. EPG is a tool used to help speech therapy processes among patients with various backgrounds such as Down syndrome, paralysed, and autism. In late 2018, Zin et al. has done a study to detect the tongue and hard palate contact for the paralysed patients [46, 47]. The researcher faced difficulties, particularly during the recording procedure. The EPG used for the research considered not user-friendly for patients who had limited physical movement. Therefore, they propose a more advanced EPG technology that may prevent the patient from being bogged down by the wires, while allowing them to record continuous speech patterns in a very comfortable condition. A smart system that incorporates the EPG enables the user to explore the benefit anywhere and everywhere. Additionally, the artificial palate can be customized matching patient's need for either therapy, learning or monitoring procedure.

EPG has now become well established in many experimental phonetic laboratories and speech. Most of the problems associated with the technique in the early stages of EPG development include unwanted capacitance, effect between closely bunched wires, saliva bridging of adjacent electrodes, and suitable material for the palate [3]. Further improvement of the EPG device is ongoing to ensure the system is safe, robust, and incorporate the latest or new technology. EPG can be upgraded and incorporates current technology in the medical field, such as Bluetooth technology, telemedicine, and mobile application.

The primary purpose of utilizing the latest technology is to transfer data from patient to computer or from one computer to another computer. Estimating by 2020, there will be more than 50 billion medical devices will be connected to the internet using wireless technology. This is because wireless technology can reduce operating costs and offer low data at minimal power for wireless sensors and actuators network applications [48].

A new architecture for the technological development of EPG is also proposed in this paper. The architecture of the advanced EPG system can be classified into two parts: the development of the hardware system and the development of the software system. Additionally, the hardware consists of the EPG palate and an embedded electronic circuit.

**Fig. 6:**
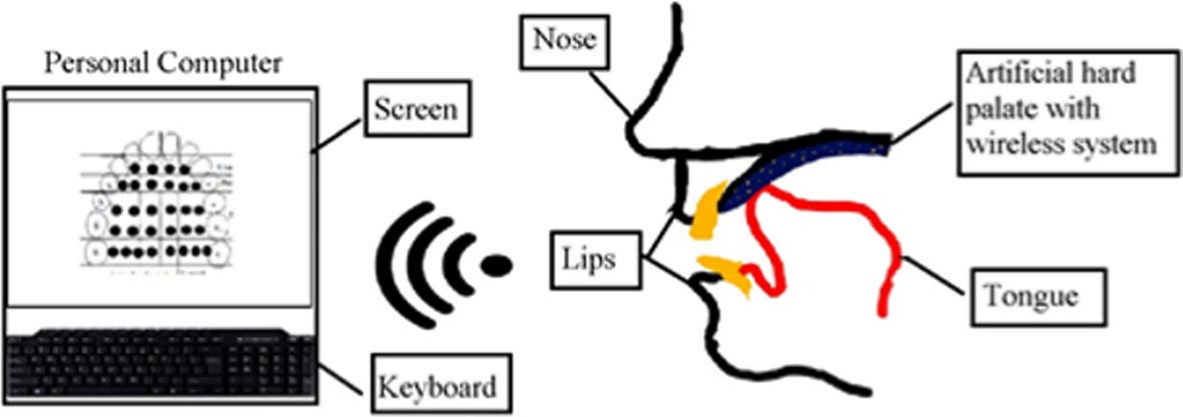
The diagram of the advanced technology of EPG

During data collection, the subject will be asked to place the EPG palate on an upper palate inside the mouth. Electrodes will be scanned by the electronic circuit, and the presence of the electrodes signal will identify tongue–hard palate contact. The electrodes signal will be transmitted to the computer for display, storage, and analysis via Bluetooth communication. The electrodes signal will be processed in the computer and presented as a meaningful contact pattern data. The tongue and hard palate contact will be displayed on the computer in real-time and thus provide real-time feedback and analysis to the user and speech therapist. Figure 6 shows the diagram of the advanced technology of EPG.

**Fig. 7:**
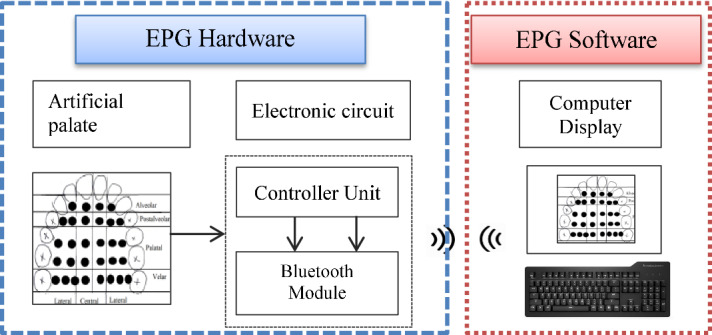
The component of the advanced technology of the EPG system

In the Reading system, the Reading palate is soldered to a connector board and will be plugged into a board reader called multiplexer in the EPG3 system. The multiplexer is plugged to the main unit, and data is transferred to a computer. The multiplexer is hanging around the subject's neck during data recording, and the subject cannot move freely [4]. Additionally, the hanging multiplexer may cause discomfort to the patients. The new technology of EPG will tackle this issue by adopting wireless technology to transfer the contact signal data during the articulation in real-time. The EPG sensors are soldered to the microcontroller board and attached to a headset. There will be no wire hanging around the patient’s neck during the speech recording. Figure 7 shows the parts of the advanced technology of the EPG system.

Besides adopting wireless technology, another suggestion is improving the artificial palate. As known, artificial palate with an embedded electrode was used to detect the contact between the tongue and hard palate during the articulation. However, an advanced EPG palate can be upgraded in terms of the contact sensor’s design—for example, the quantity, size, and location of the silver electrodes.

This study suggests the new design of artificial palate, which consists of 30 silver electrodes in five horizontal rows. The first row contains four electrodes, second to fourth row includes six electrodes, and the last row comprises eight electrodes. The arrangement of the first row starts right after the incisor teeth, and it is known as the alveolar area. The post-alveolar area is behind the bicuspid teeth. Meanwhile, the palatal area consists of third and fourth rows, which starts between the first and second permanent molar teeth. The fifth row is the velar area that begins at the third permanent molar teeth. More importantly, the proposed design can be customized based on the needs of the user and the function of the EPG palate, either for therapy, monitoring or learning. Hardcastle et al. [4] stated that the number of electrodes depends on the study’s objective, such as in their research, which involve children who have smaller palate size, one or two rows electrodes were eliminated. This statement was also supported by Flege et al. [49], which arranged 64 electrodes in six rows for the productions of /*s*/ and /*t*/. Besides, the previous artificial palate, such as the Reading palate consists of 62 silver electrodes [4], the Rion system consists of 63 gold electrodes [3], and Kay Palatometer system consists of 100 gold electrodes [10]. Hence, it is highlighted that articulation places are more critical compared to the number of electrodes being placed on the artificial palate.

In addition, the new design of artificial palate suggests a new size of electrodes which is the diameter of the electrode is 4 mm, and it is soldered to a 20 cm length of copper wire. The purpose of this enlargement is to ensure the electrode is able to fill up the palate zones. Hardcastle et al. [18] in their study stated that the average time in the process of manufacturing the Reading palate started from dispatch of plaster impression to the delivery of the Reading palate were estimated as eight days per palate. Thus, by reducing the quantity of the materials, the manufacturing cost will also be reduced.

## Fabrication method

**Fig. 8:**
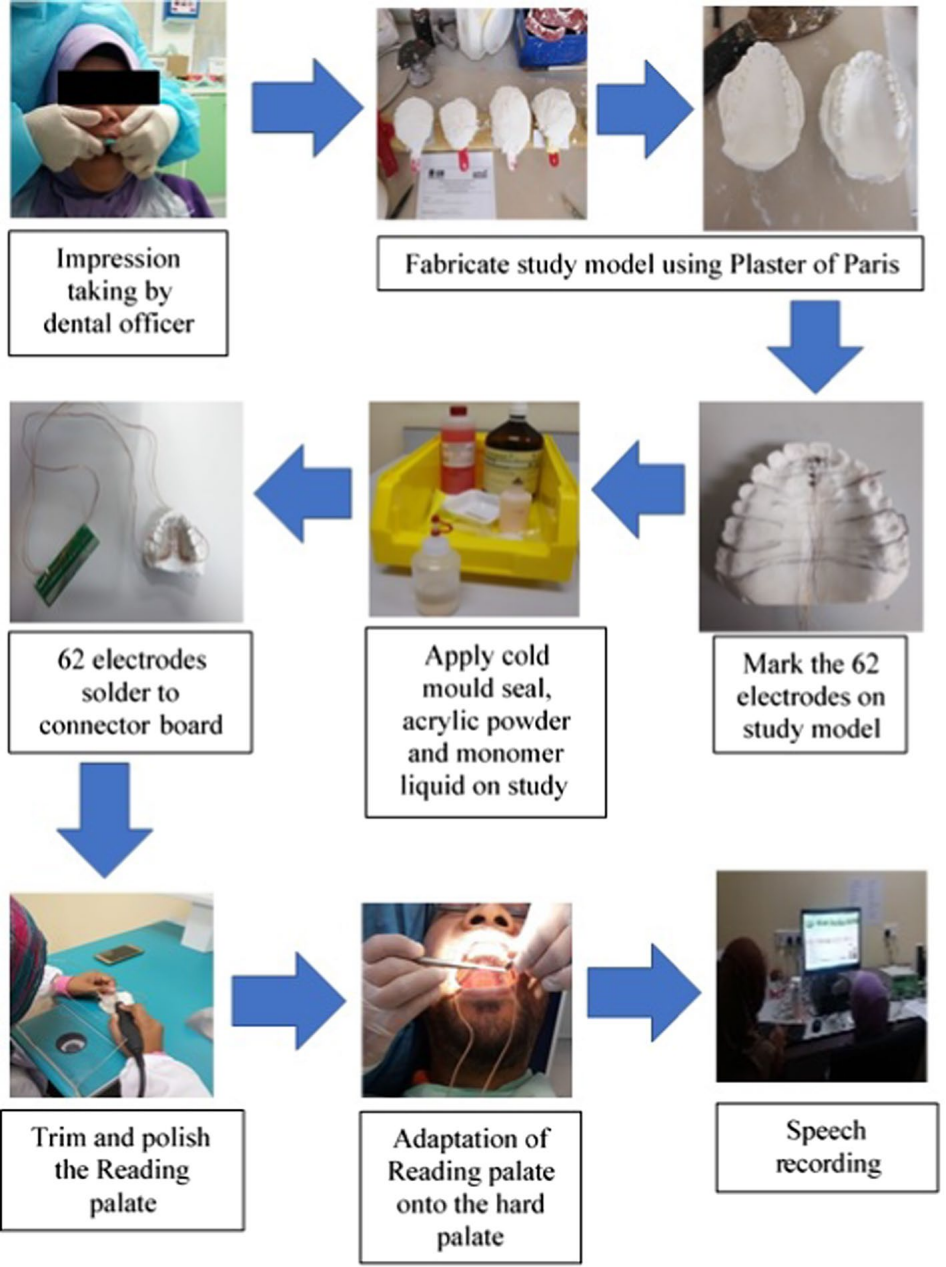
The fabrication process of the artificial palate from impression taking until speech recording

Fabrication of EPG’s advanced technology is divided into three parts, which is the fabrication of the EPG palate, development of the electronic circuitry, and development of the software interface. The fabrication of the advanced technology of EPG palate is similar to the manufacturing of a Reading palate. Starting from taking an upper arch impression to the adaptation of the EPG palate onto the hard palate, as shown in Fig. 8.

The advanced EPG electronic functioned to detect the tongue and hard palate contact during speech and transfer the signal to the computer through Bluetooth communication. The circuit consists of electronic components such as a microcontroller, capacitor, silver electrodes, MHz Crystal oscillator, and a Bluetooth system. The microcontroller is the most important electronic component, which acts as the brain of the electronic circuit. The microcontroller defines the input pins and the output signals. The microcontroller processes the analogue signals received by each of the electrodes embedded on the artificial palate. Other electronic components, such as a capacitor, crystal oscillator, and several others, are required to support the microcontroller.

The software component is categorized into the front-end and back-end. The front-end includes a graphical user interface (GUI) that allows users to interact with the advanced EPG system through a graphical icon. GUI also enables data to be displayed in real-time. Besides, the GUI is designed to record the contact during the production, save the contact data to the computer, and allows contact pattern data to be further analysed.

The contact pattern between the tongue and hard palate data from the microcontroller is transferred to a computer through a Bluetooth module. GUI can also be designed to access the contact pattern data using a COM port connection. The back-end of the software is the program written to process the signals into meaningful data. The program may also include signal processing options such as filtering, amplifying, and standard referencing. The program can also be used to run analysis and command based on user selection in the GUI.

## Important characteristics

**Fig. 9:**
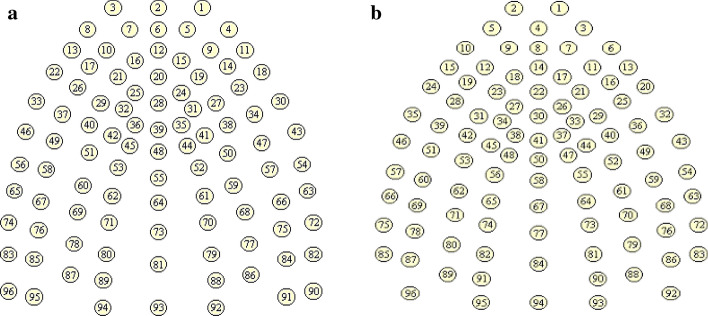
Default layout **a** English speaker and **b** French, Korean and Taiwan speakers (reproduced with permission by Keating [51])

Based on the selected study, an important characteristic should be incorporated to design and develop new EPG technology. It is vital to ensure that EPG's future technology becomes more convenient for patients with limited movement capability. This suggestion was highlighted by Wrench et al. [3], and some are matched with the report by Hardcastle et al. [4]. The requirement and suggestion are:

1. Thickness: thin palate allows the user to pronounce such a normal speech to obtain an accurate result without any interference.
2. The number of contacts: the number of contacts varies from 62 to 124 electrodes. In comparison, there are more disadvantages to produce a larger number of connections. The cost will be higher, and the electrode coverage is difficult to distinguish between singleton since the contact pattern is almost similar [50].
3. Robustness: palate must be robust enough to use multiple times without changing in shape, although the palate is thinner.
4. Contact size: the smaller the contact diameter, the more accurate the contact position. However, conductivity will be lower.
5. Contact material: the tarnish factor is critical because the application used in the mouth is exposed to the saliva. Either gold or silver, both are a good conductor. However, silver is preferable since the palate will be used in a short period.
6. Flexible circuit material: polyimide is usually used as the base insulative layer, while the copper conductor often is plated either with gold or silver. Silver plating is preferable since the process does not use the poisonous chemical and silver-loaded epoxy to coat the silver plate contact.
7. Safety: the use of nontoxic materials is essential to ensure no chemical reaction will harmful the user. Acrylic resin is the common dental base. Acrylic resin is used to cover the flexible circuit and simultaneously provide a smooth surface and minimize sharp edges.
8. Adjustability: the artificial palate should fix or accommodate to small movement during the speech treatment. The shape also must be usable for various ages, especially for children, which grows within the treatment time.
9. Target patient or user: every language tends to control different tongue movement and position during pronunciation. Therefore, the electrode must be placed in the right spot. For example, Keating et al. suggest placing two electrodes in the middle of the front two incisors for French, Korean, and Taiwan speakers as compared to English speakers [51], as shown in Fig. 9.

Further detailed comparison of the artificial palate between Kay Palatometer, Reading, CompleteSpeech Palatometer, and Articulate palate is shown in Table 2.

**Table 2:** Comparison of the three types of the palate in different aspects

Factor consideration	Kay palatometer system		Reading system		CompleteSpeech palatometer		Articulate system	
Picture					Not available		Not available	
Number of contact	96 (copper gold plated electrode)		62 (thin silver discs electrode)		124 (gold plate electrode)		62 (silver plating electrode)	
Diameter of contact	1-mm-diameter gold toroids Gold also is a good conductor and less susceptible to tarnish		1.4-mm-diameter silver discs Silver is a good conductor, but it is known to tarnish over extended periods of time when exposed to sulphides, which can be found in the air and saliva		Not mentioned		1.5-mm-diameter through the palate Using silver plating of circuit contacts and less toxic chemical of processing	
Thickness of palate	1 mm		1.5–2.5 mm		~ 0.5 mm		~ 1 mm over the teeth	
Coverage area	Moulded to fit the speaker’s hard palate and to cover the external border of the upper teeth Goes further back in the mouth toward the soft palate (up to the back of the molars), particularly in the mid-sagittal plane		Covers only the hard palate and stops at the gingival border		Coverage on the velar and dental region		More coverage on velar and dental regions The electrode position at the velar region is almost similar to Kay palate coverage, 7–12 mm behind the border of the hard and soft mid-sagittal	
Price and cost (see [3, 48], pricing according to manufacturer inquiry)	High cost by the usage of gold and a greater number of contacts USD 400		Estimated cost USD 570 for traditional Reading palate [43]		USD 300 not including dental impression cost		USD 320 for a new fully compatible Articulate Palate [43]	
Electrode placement	Location of electrode	Number of contact assign	Location of electrode	Number of contact assign	Location of electrode	Number of contact assign	Location of electrode	Number of contact assign
	Dental region	8	Dental region	0	Dental region	12	Dental region	6
	Alveolar region	11	Alveolar region	14	Alveolar region	24	Alveolar region	8
	Post-alveolar	16	Post-alveolar	16	Post-alveolar	1414	Post-alveolar	16
	Palatal region	47	Palatal region	24	Palatal region	42	Palatal region Palatal region	16
	Velar region	14	Velar region	8	Velar region	32	Velar region	16
Method of manufacturing	1. A dentist prepares the stone cast of the user by obtaining a plaster dental impression of the upper jaw 2. 96 gold contacts are embedded between the top and base layer of the acrylic vacuum and cover the teeth area 3. Each contact is soldered with copper wire, grouped at the left and right side and exiting behind the rear molars 4. Using a flexible tube, the wires are sealed and soldered to pin connector plug		1. Almost similar to Kay Palatometer manufacturing, a dentist is required to take an impression of the upper jaw, and a plaster model is prepared based on the impression 2. The electrode position is marked on the surface of the plaster model with an indelible pencil 3. 62 of silver contact are then embedded in acrylic resin 4. Stainless steel Adams clasps clip is applied to ensure the palate retains in place during the treatment 5. Fine copper wires are soldered on each of the contacts, grouped and exiting behind the rear molar as similar to Kay Palatometer 6. Using a flexible tube, the wires are sealed and soldered to a double-sided edge connector card		1. A dental impression is required or scanning a 3D image of the mouth/upper palate also available, which then access to a 3D printer to prepare the dental mould 2. The SmartPalate is then created based upon the dental mould and thermoformed using soft plastic 3. A flexible printed circuit is attached after cutting and conforming the shape and fit the user 4. The flexible circuit is connecting to processing and display equipment by leads containing electrical connectors		1. Silver-loaded epoxy compound bonded onto each of silver plate contact 2. The circuit then sealed between two layers of acrylic 3. Wires exit the mouth at the front teeth	
Time manufacturing	Not mentioned		Palate manufacture generally takes between 12 and 16 h but can take longer if wires are accidentally damaged while the palate surface is polished		Completed within 5 business days from the date the stone model was received		Take approximately 3–4 h	
Adjustable fit	Thermoformed palate covers the teeth		Using acrylic resin and Adams clasps		Thermoformed palate covers the teeth		Compounds of wax and ethyl vinyl acetate (EVA) is used to fit into the mouth The wax and EVA are softened when placed in hot water. After the user wears the palate, it flows between the teeth and solidifies	

The primary purpose of developing the advanced EPG system is to provide a more convenient device for the end-user. The new EPG design must consider a few characteristics such as cost-effectiveness, comfort for the user, and material safety. Table 3 shows the features and the description of the advanced EPG system.

**Table 3:** The critical features of the advanced technology of the EPG system

Features	Description
Safety	The materials used in developing advanced EPG is nontoxic and biodegradable. The materials used for the artificial palate are acrylic resin, silver electrodes, and copper wire. Acrylic resin has been approved by the FDA and is widely used in dental applications such as retainer and denture. In addition, silver electrode and copper wire are also widely used as sensors in a medical application such as EEG and EMG. Besides, the electronic component used in the development of new EPG must prevent electric shock
User friendly	During data recording, the multiplexer is hanging around the subject's neck, and the subject cannot move freely. The design of new EPG is more friendly to the environment and the user. The use of Bluetooth technology may avoid the need of the patient from being bogged down by the wires, and the patient can record continuous speech patterns in a very comfortable condition. The other advantage of the new EPG is saving the workspace area compared to the previous EPG, which needs a large workspace to place a personal computer, sound system, microphone, and main unit of the EPG. The new technology EPG is a portable unit, and reasonably small space is needed during the recording
Cost-effectiveness	Hardcastle et al. [18] in their study stated that the average time in the process of manufacturing the Reading palate started from dispatch of plaster impression to the delivery of the Reading palate was estimated as 8 days per palate. It may affect the labour cost and time. The new EPG may reduce the manufacturing cost and time by reducing the manufacturing process and materials used

## Conclusion

EPG has been found useful in both diagnosis and rehabilitation of the range of the speech disorder. The primary purpose of EPG is to detect the contact pattern of the tongue and hard palate. However, further improvement of the EPG device is ongoing to ensure the system is safe, robust, and incorporates the latest technology available in the market. EPG can be upgraded and incorporates current technology in the medical field, such as Bluetooth technology, telemedicine, and mobile application. Three additional features have been suggested for improving the EPG system in the future, including safety, user-friendly, and cost-effectiveness. However, the improvement may be expanded further and not limited to these three features explained in this review. Combining the latest technology to the EPG system allows transferring data from users to therapists and vice versa. Additionally, the cloud system can also be introduced to store data and easily share medical data among therapists. Simultaneously, real-time monitoring becomes possible and hopefully will ensure effective speech treatments for the patients.

## Data Availability

Not applicable.

## Abbreviations

COM: Communication port
EPG: Electropalatography
GUI: Graphical user interface
SLT: Speech–language therapist
SPI: Serial port interface
SSD: Speech sound disorder
TPTD: Tongue position tracking device
USA: United States of America
USD: United States dollar
